# Optically-active metastable defects in volumetric nanoplasmonic composites

**DOI:** 10.1038/s41598-018-30803-0

**Published:** 2018-09-07

**Authors:** Marcin Gajc, Hancza B. Surma, Dorota A. Pawlak

**Affiliations:** 1 Institute of Electronic Materials Technology (ITME), Wolczynska 133, 01-919 Warsaw, Poland; 2 Chemistry Department, University of Warsaw, ul. Pasteura 1, 02-093 Warsaw, Poland

## Abstract

Metastable defects in semiconductor materials have been well known for decades, but have only recently started to attract attention for their potential applications in information technology. Here, we describe active and passive nanoplasmonic materials with optically active metastable defects that can be switched on or off by cooling with or without laser illumination, respectively. To the best of our knowledge, this is the first report of metastable defects in either passive or active nanoplasmonic materials, and, more generally, in non-semiconducting materials. The nanocomposites are made of a sodium-boron-phosphate glass matrix doped with silver nanoparticles (nAg) or co-doped with nAg and Er^3+^ ions by NanoParticle Direct Doping method. We further show that the different origins of the two types of defect-related luminescence behaviour are attributable to either a metal-glass defect (MG1) or a metal-glass-rare-earth ion defect (MGR1). Such materials could potentially be used for data writing and erasing using laser illumination with a ‘tight’ focus such as direct laser writing.

## Introduction

Metastable defects occur when at least two different atomic arrangements are possible for a given defect charge state. The different arrangements are separated by an energy barrier where one is more stable than the other, less stable or metastable defect^1^. The most well-known metastable defect is EL2 (arsenic antisite), a native deep-donor defect in GaAs^2–5^, but such defects have also been found in GaN epilayers^6,7^ and other semiconductors such as silicon^8^ and InP^9^. Defects can participate as radiative or non-radiative recombination centres, which parasitically affect the luminescence intensity of important optical processes. As such, metastable defects make important contributions to the spectroscopic properties of semiconducting materials.

Recent studies have started to examine the use of metastable defects for various novel applications, such as memory cells (volumetric optical storage)^10–12^, switches and other nonlinear devices^13^. In the last few years, the reading of data stored in metastable defects in silicon has been demonstrated^10^ using light for both reading and writing of information in three-dimensions, without the need for complex three-dimensional electronic interconnections. This type of three-dimensional, or volumetric, data storage has potential benefits for improving storage density for more compact elements. However, the demonstrated bit size for storing data in metastable defects in silicon was relatively large (1 µm^2^), and the defects that enabled this demonstration were not stable over time.

Utilizing defect-related emissions for optical storage has also been demonstrated in vitreous SiO_2_ with mechanically-generated defects^14^. However, in this case, the laser applied for writing damaged the SiO_2_ structure because relatively high-energy pulses were required. Additionally annealing at a high temperature (400 °C) for a relatively long time (30 min) was required to erase the “written” defect-bits. To date, metastable defects have only been demonstrated in standard semiconducting crystalline materials, although the creation of this type of defect should also be possible in novel nanomaterial composites. The development of such composites could provide nanoscale optical memory elements that overcome the challenges of instability and damage during switching, which could therefore lead to new applications.

One of such interesting types of materials are metallodielectric nanocomposites. They offer many novel functionalities due to their unusual electromagnetic response, manifested in surface plasmon excitations being a consequence of interactions between collective electron oscillations (plasmons) and photons^15^. In plasmonic nanomaterials, nanostructuring of metallic systems enables localization of light in very small volumes with a concentration of light (fields) at the resonant frequency^16^. This phenomenon has led to novel applications, including the development of sensors^17^, plasmon-based lasers^18,19^, efficient two-dimensional optical information storage^20^, highly efficient solar cells^21^, new types of photonics devices^22^, and plasmonic photo-thermal anti-cancer therapy devices^23^.

Various methods to enable the manufacturing of such materials based on bottom-up approaches have recently been developed^24–27^. Some of these bottom-up manufacturing methods involve either chemical^28^ or non-chemical^29^ doping of glass matrices with plasmonic nanoparticles. In chemical doping, metal is typically introduced in the form of ions and the metallic nanoparticles are only formed after a long annealing process. On the other hand, in non-chemical doping, metallic nanoparticles are introduced directly into the glass matrix^29^.

Until now, very few optical studies of the defects in glasses doped with Ag nanoparticles have been conducted. These studies include the observation of various silver species in different oxidation and aggregation states, such as luminescent single Ag^+^ ions, Ag^+^-Ag^+^ and Ag^+^-Ag^0^ pairs, obtained by melting of phosphate glasses^30,31^. Some efforts have been undertaken to study the energy transfer and evolution of optical processes in glass after thermal treatment^32,33^, to investigate glass that contains Eu^3+^ ions^34^, and to analyse glass after femtosecond laser pulse application^35^ and X-ray irradiation^36^. Photoluminescence from these defects have been observed in the 200–600 nm wavelength range.

**Figure 1:**
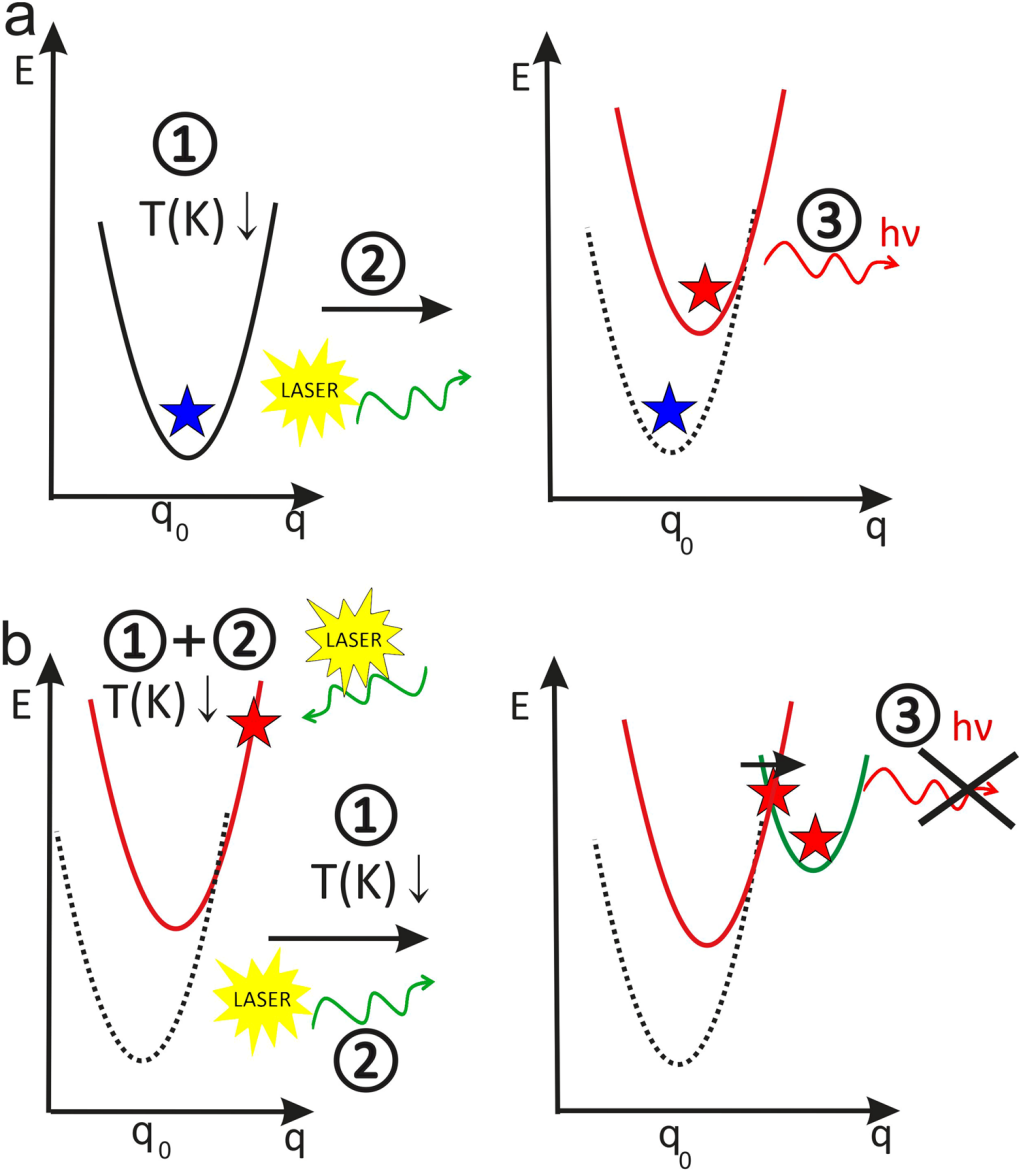
Optically active laser light and temperature-dependent on/off-switching of metastable defects in glass-metal-rare earth ion and glass-metal nanocomposites. (**a**) PL-active defect state after cooling the materials in darkness. (**b**) PL-inactive defect state after cooling the materials under laser light illumination. The curves indicate energy states for MGR1/MG1 defects vs configurational coordinate q: black curve - optically active ground state; red curve optically active excited state; green curve - metastable state. Processes: 1-cooling down; 2-illumination with laser beam; 3-emission/no emission from defect.

Here, we demonstrate optically active defects in passive and active bulk nanoplasmonic volumetric materials, which exhibit emission in the near-infrared wavelength range. The bulk nanoplasmonic materials made of sodium borophosphate glass (Na_5_B_2_P_3_O_13_, NBP) doped with silver nanoparticles or co-doped with silver nanoparticles and erbium ions exhibit defect-related low-temperature photoluminescence. This luminescence can be switched off by cooling the samples under laser light illumination in darkness and switched on by cooling the samples in darkness without laser light illumination, Fig. 1. To the best of our knowledge, this is the first time that metastable defects have been demonstrated in a nanoplasmonic material, and more generally, in a non-semiconducting material.

## Defect luminescence in volumetric nanoplasmonic materials

**Figure 2:**
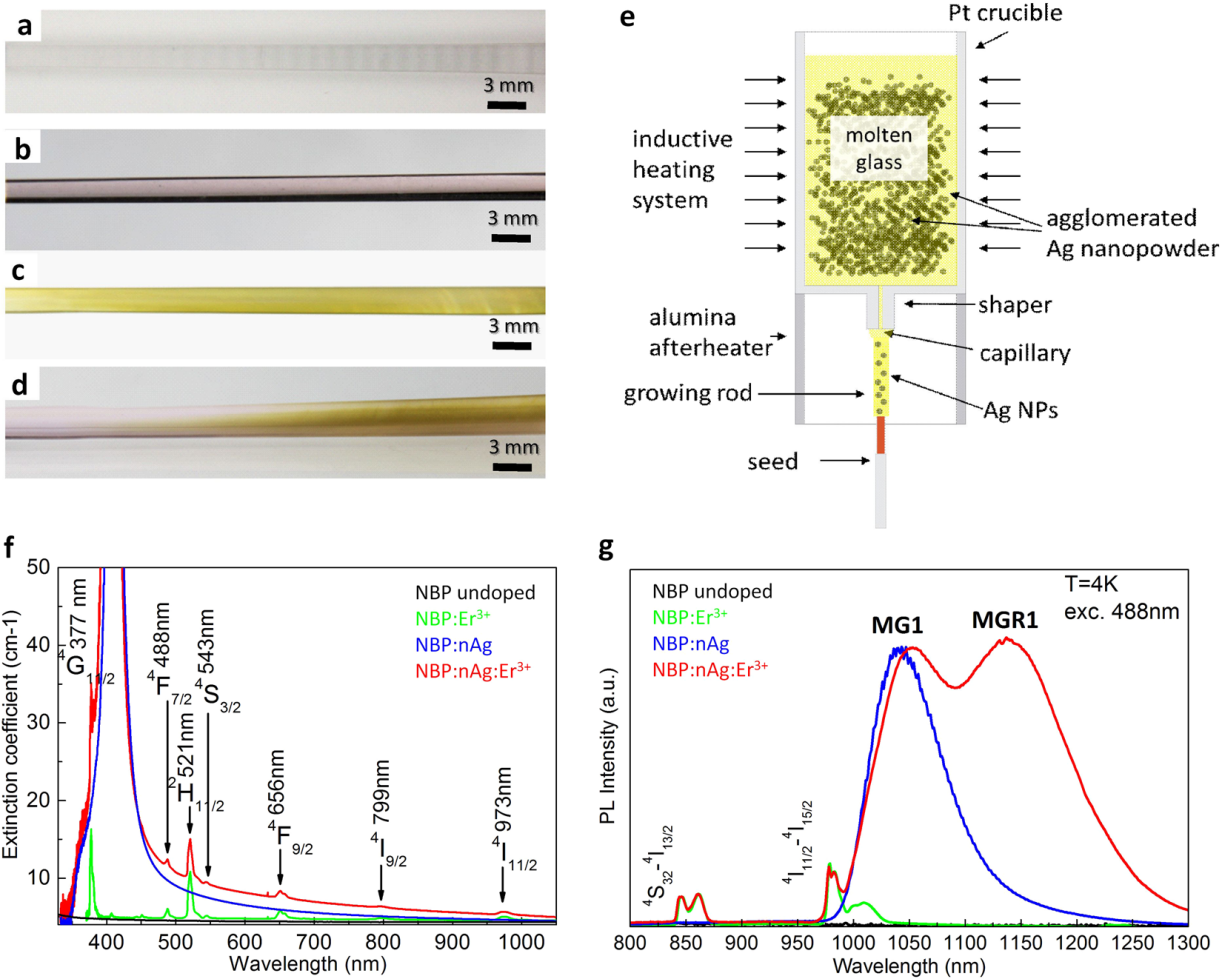
Passive and active bulk nanoplasmonic materials obtained by the NanoParticle Direct Doping (NPDD) method^1^ that demonstrate low-temperature defect-related photoluminescence. (**a**) Undoped NBP glass rod. (**b**) NBP rod doped with erbium ions (NBP:Er^3+^). (**c**) NBP rod doped with Ag nanoparticles (NBP:nAg). (**d**) NBP rod co-doped with Ag nanoparticles and Er^3+^ ions (NBP:nAg,Er^3+^). (**e)** Scheme of NPDD, where plasmonic nanoparticles and optically active elements, such as rare earth ions, are added directly to dielectric matrices. (**f**) Room temperature extinction coefficient of NBP glass-based nanocomposites, which demonstrate plasmonic resonance at ca. 400 nm and Er^3+^ absorption lines. (**g**) MG1 defect-related low-temperature PL observed in NBP:nAg, and MGR1 defect-related low-temperature PL observed in NBP:nAg and NBP:nAg, Er^3+^ samples.

Bulk nanoplasmonic materials were obtained by the NanoParticle Direct Doping (NPDD) method, in which sodium-boron-phosphate, Na_5_B_2_P_3_O_13_, glass was used as a matrix for embedding silver nanoparticles (nAg) and/or erbium (Er^3+^) ions, Fig. 2a–d. NPDD^29^ enables the direct doping of dielectric materials with various nanoparticles and co-doping of the nanoparticles with other chemical species such as rare-earth ions, without the need for chemical reactions, Fig. 2e. Using NPDD, we recently demonstrated a 7-fold enhancement of photoluminescence in the composite, which was co-doped with silver nanoparticles and erbium ions, in comparison to the sample without plasmonic nanoparticles^29^.

The obtained bulk materials doped with silver nanoparticles, NBP:nAg and NBP:nAg,Er^3+^, show strong extinction bands with a maximum at 405 nm, which is attributable to the localized surface plasmon resonance (LSPR), Fig. 2f, as has been demonstrated previously^29^. The strong LSPR band indicates the presence of relatively large amounts of nAg, while its symmetric shape confirms that the 20 nm diameter nanoparticles used here fall within the size limit of ‘small particles’^37^, and thus produce limited scattering. The extinction spectra of the materials doped with Er^3+^ ions, NBP:Er^3+^ and NBP:nAg,Er^3+^ exhibit also the typically narrow features associated with 4f-4f electronic transitions from the ^4^I_15/2_ ground state to various excited Er^3+^ states^38^.

The manufactured materials exhibit low-temperature defect-related broad photoluminescence when doped with nAg, with a maximum band at 1050 nm (MG1 – the metal-glass defect), and an additional band at 1150 nm (MGR1 – the metal-glass-rare-earth ion defect), when the materials are co-doped with nAg and Er^3+^ ions. This is shown in Fig. 2g for the photoluminescence (PL) spectra of NBP:Er^3+^, NBP:nAg, and NBP:nAg,Er^3+^ samples measured under excitation with a 488 nm laser line at 10 K. The sharp emission lines at approximately 860 nm and 980 nm in the erbium-doped nanocomposites were identified as ^4^S_3/2_ – ^4^I_13/2_ and ^4^I_11/2_ – ^4^I_15/2_ Er^3+^ ion transitions, respectively^32^.

## Temperature dependence of the defect photoluminescence

**Figure 3:**
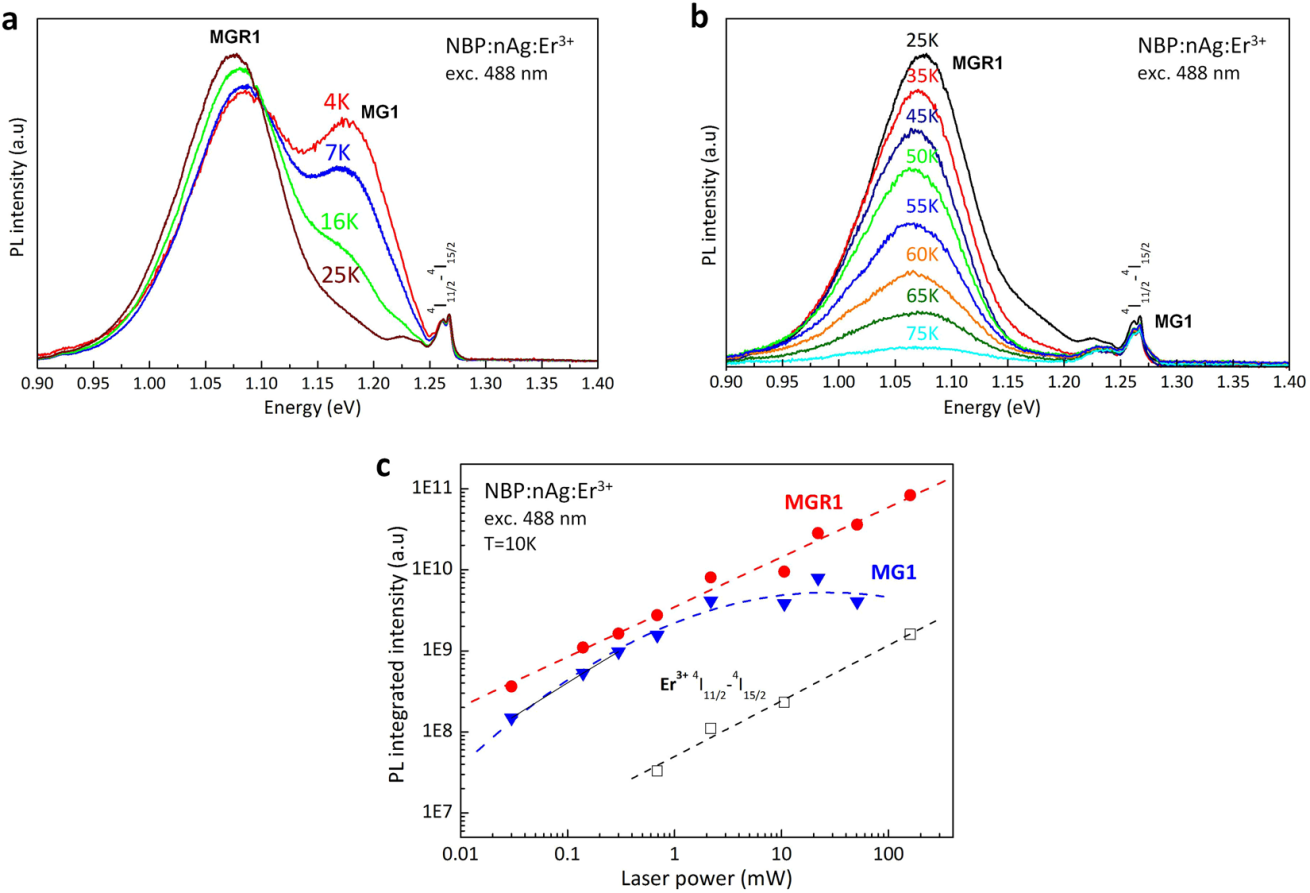
Different origins of the two observed metastable defects confirmed by differences in the dependence of the photoluminescence intensity on temperature. (**a**) PL spectra at 4 K–25 K, and (**b**) PL spectra at 25 K–90 K, which demonstrate the different behaviours of the MGR1 and MG1 defect-related photoluminescence. (**c**) Dependence of the integrated intensity on the excitation power for the MGR1 and MG1 bands in comparison with the ^4^I_11/2_–^4^I_15/2_ electric-dipole transitions within the 4 f shell of Er^3+^.

The difference in the evolution of the defect-related emission with temperature for the NBP:nAg,Er^3+^ and NBP:nAg samples suggests that both MGR1 and MG1 emissions are related to two different luminescence centres with different microscopic natures. When the samples were heated, the MGR1 and MG1 bands disappeared at different temperatures (above 90 K and at 30 K, respectively), Fig. 3a,b.

That two different centres are responsible for the emissions at 1150 and 1050 nm is also indicated by the different concentrations of defects responsible for the MG1 and MGR1 emissions. For the applied power range, the MGR1 emission intensity at 1150 nm and the Er^3+ 4^I_11/2_–^4^I_15/2_ transitions show a linear dependence on the excitation power, while the MG1 emission starts to saturate for a laser beam power higher than 1 mW, Fig. 3c. It has been found experimentally that the relation between MGR1 emission intensity, I_MGR1_, and the emission from erbium ions, I_4I11/2-4I15/2_, is described by the relation I_MGR1_ = 8.14 * 10^10^ + I_4I11/2-4I15/2_. This indicates that the concentration of MGR1 defects can be directly related to the concentration of erbium ions. In contrast, the MG1 emission is related to the presence of silver nanoparticles, and the concentration of MG1 defects should be proportional to the concentration of nAg. It has to be emphasized that there is no correlation between the formation of MG1 defects and erbium ions since they are also present in the samples doped only with silver nanoparticles (NBP:nAg). The saturation of the power dependent MG1 emission suggests lower concentration to MG1 defects for excitation power higher than 1 mW can suggest lower concentration of MG1 defects than MGR1 ones. This is possible only if more than one erbium ion interacts with a silver nanoparticle, which is extremely likely, since nAg can be treated as a large ‘precipitate’ (~20 nm in diameter) compared to Er^3+^ ions (~0.1 nm in diameter) in the NBP network.

## Metastable defect switching

**Figure 4:**
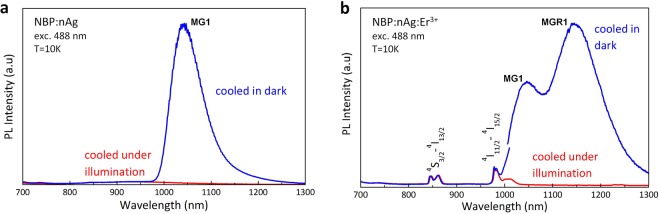
Optically active metastable defects in active and passive bulk nanoplasmonic materials. (**a**) PL spectra for sample doped with silver nanoparticles (NBP:nAg) (**b**) PL spectra for sample co-doped with silver nanoparticles and Er^3+^ ions (NBP:nAg,Er^3+^). The blue curves represent the PL spectra after cooling the sample without illumination; the red curves represent the PL spectra obtained after photo-quenching of MGR1 and MG1 emissions by illumination with a laser beam during the cooling-down process.

The discovered MGR1 and MG1 defect-related luminescence identified above can be switched off by cooling the samples under laser light illumination and switched on by cooling the samples in darkness. When the NBP:nAg,Er^3+^ and NBP:nAg samples were cooled from room temperature to 10 K under 488 nm laser beam illumination, the MGR1 and MG1 peaks disappeared, Figs 1b and 4. However, they can easily be thermally recovered after heating up the samples to room temperature and then cooling them down in darkness. No significant differences were observed in the extinction spectra measured at 15 K for the NBP:nAg,Er^3+^ sample cooled in darkness and the NBP:nAg,Er^3+^ sample cooled under illumination, Fig. S1. This suggests that the excitation of the MGR1/MG1 defects from the ground to the excited state occurs directly by the absorption of the laser energy or because of the energy transfer from erbium ions or silver nanoparticles to MGR1/MG1.

As it has been already demonstrated previously metallic nanoparticles can heat efficiently under electromagnetic radiation, and especially when the irradiation is at the range of the plasmon resonance^39^. Heat generation by the nAg in our materials potentially could lead to switching off the MG1 emission when the sample is cooled under in the presence of laser light. Govorov and Richardson^39^ have shown the dependence of the temperature increase at the surface of a single Au nanoparticle on the irradiation power density at the plasmon frequency. The significant temperature changes occur when the particles are bigger and at the power densities of 10^2^–10^5^W/cm^2^. In our case the laser spot was of 0.3 mm diameter and of S = 7.06 * 10^−4^ cm^2^ surface. The maximum intensity of the laser beam was 180 mW that gives the power density around 255 W/cm^2^ so the highest power that can be absorbed by a single silver nanoparticle 20 nm in diameter is equal to 8 * 10^−4^ μW. However the energy absorbed by silver nanoparticle in our material is even lower as the excitation with 488 nm lies pretty far from the maximum absorption at 405 nm for silver nanoparticles. Comparing with the example provided^39^ for 20 nm diameter single Au nanoparticle and the light spot of 2 μm diameter from the flux around 200 W/cm^2^ the temperature increase is lower than 0.05 K. For Ag it can be 10 times larger but still we do not expect that heat generation plays a role in the switching on and off the defect emission in the presented here materials.

## Characterization of defect centres

The metastable and bistable behaviour of the defect can be explained using the configurational coordinate (CC) model^40^, which assumes that the energy of the defect or impurity depends on its atomic configuration. The CC model enables the analysis of the total energy of the ‘defect + lattice’ system in different charge states, such as the ground state and excited states. The model can also be applied to explain the behaviour of defects that are able to change their atomic configuration under pressure, illumination or temperature. A more detailed description of the model has been included in the Supplementary Materials.

**Figure 5:**
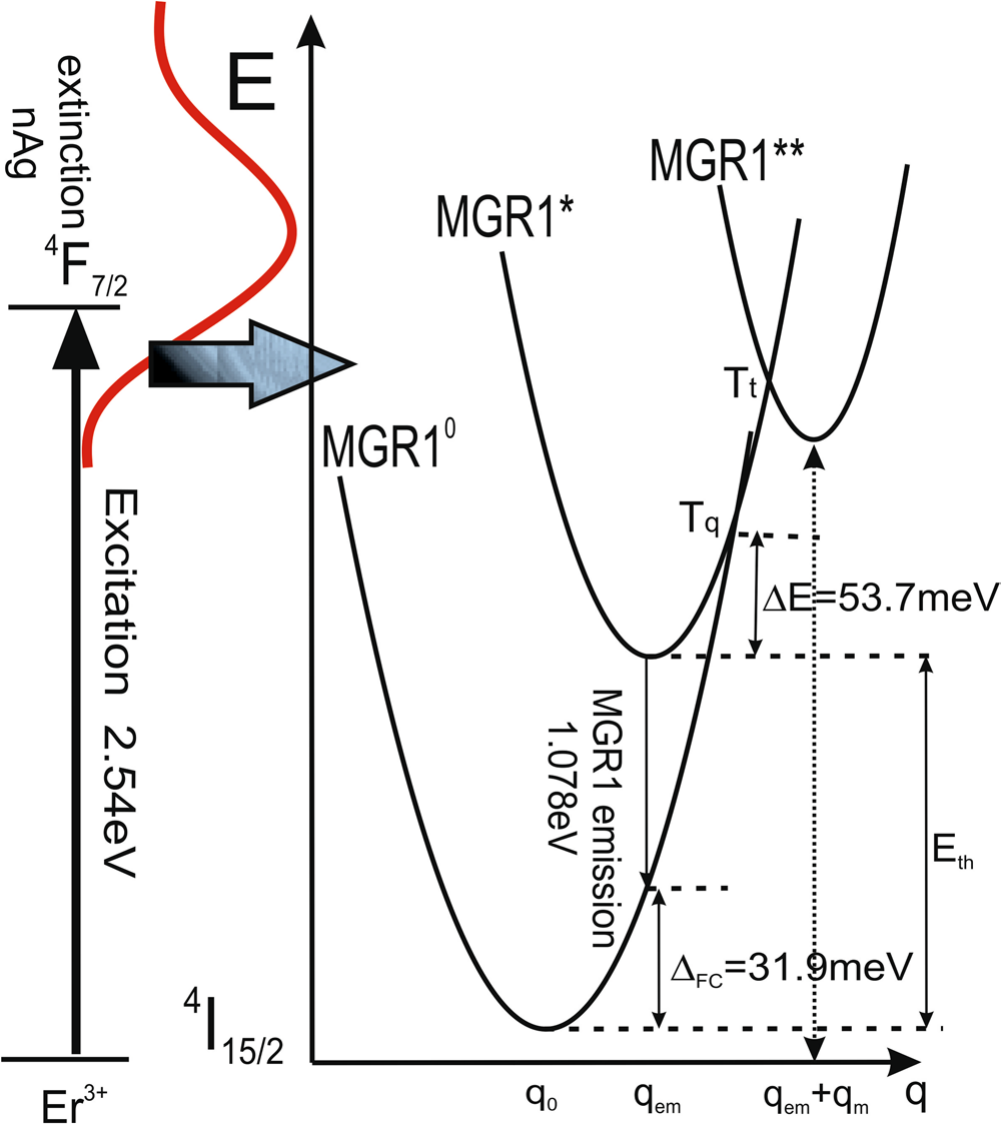
The configurational coordinate (CC) diagram for the MGR1 defect centre, based on the experimental photoluminescence results. A CC model enabled analysis of the total energy of the ‘defect + lattice’ system in different charge states, such as the ground state and excited states. q – configurational coordinate, q_0_ – q value for the ground state, q_em_ + q_m_ – q value for the metastable state of the defect, q_em_ – q value for the metastable state of the defect, q_m_ – represents the change of q value during transformation from the exited state to the metastable state, Δ_FC_ – Frank-Codon shift energy, T_t_ – defect transition temperature, T_q_ – quenching temperature, E_th_ – thermal activation energy, ΔE – activation energy.

MGR1 and MG1 are two different defects but show similar metastable behavior shown schematically in Fig. 1. Although the optical features of the MG1 and MGR1 emissions are very similar the former do not follow the CC model parameters and the construction of a CC diagram for the MG1 centre was not possible. The proposed CC diagram for the MGR1 defect centre, including the different charge states (the ground state, MGR1^0^, excited state, MGR1*, and a metastable state, MGR1**), is shown in Fig. 5. The parameters used to create the model were calculated from the PL results using the integrated intensity, the-full-width-at-half-maximum (FWHM) of the emission and the maximum position of the PL peak.

**Figure 6:**
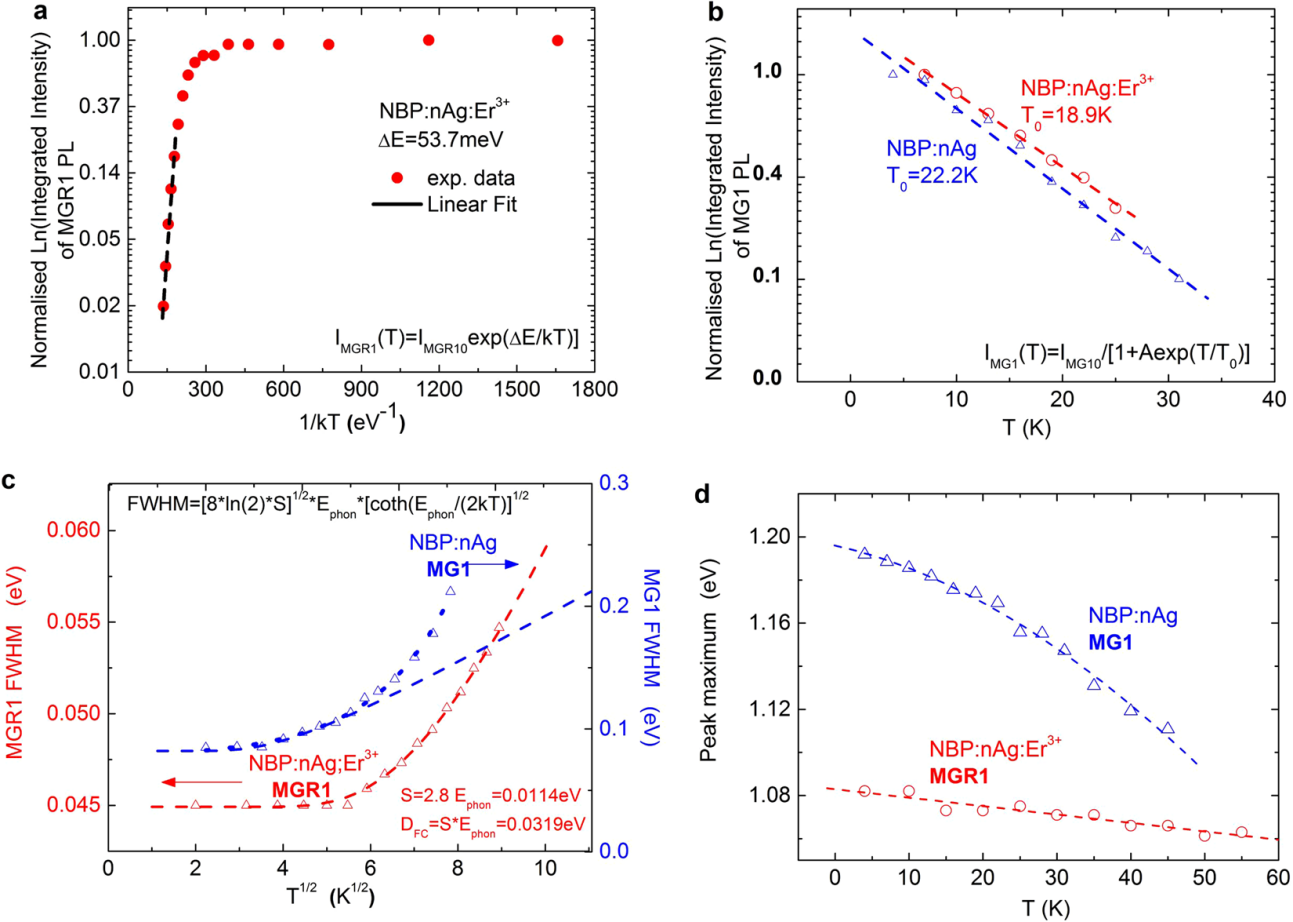
Differences between MGR1 and MG1 defect centres highlighted by the differences in the dependence of their luminescence on temperature. (**a**) Arrhenius plot of MGR1 defect-related PL *vs*. 1/kT for NBP:nAg:Er^3+^. (**b**) Linear plot of MG1 defect-related PL *vs*. temperature for NBP:nAg:Er^3+^ and NBP:nAg. (**c**) Variation of the full-width-at-half-maximum (FWHM) of the MGR1 and MG1 emissions with the square root of the temperature (triangles). Dashed curves are theoretical fits according to the configurational coordinate model. The dotted curve represents the exponential fit. (**d**) Thermal shift of the MGR1 and MG1 emission peak energy towards lower energy values as the temperature increases.

The activation energy of the 1150 nm MGR1 emission band for the thermal quenching process between 25 and 75 K (MGR1* → MGR1^0^) was calculated from the plot of the MGR1 photoluminescence intensity versus 1/kT for NBP:nAg,Er^3+^, and found to be *ΔE* = 53.7 meV, Fig. 6a. The change in the integrated intensity of the MGR1 band, normalized to the low temperature limit, was fitted with the Arrhenius formula:

1 $${{I}}_{{MGR}1}({T})={{I}}_{0MGR1}\,\exp ({\rm{\Delta }}{E}/{kT})$$

where *I*_*MGR1*_*(T)* and *I*_0*MGR1*_ are PL intensity values at temperatures T and 0 K, respectively.

In contrast to the MGR1 emission, the change of the integrated intensity of the MG1 emission at 1050 nm versus temperature could not be fitted with an Arrhenius plot. Instead, it follows the relation given for amorphous materials^32^ with localized states^33^:

2 $${{I}}_{{MG}{\rm{1}}}({T})={{I}}_{0{MG}{\rm{1}}}/[1+{A}\exp ({T}/{{T}}_{0})]$$

where *I*_*MG1*_*(T)* is the emission intensity at temperature *T*, *T* is the measured temperature, *T*_*0*_ is the characteristic temperature that corresponds to the energy depth of the localized state, A is the tunnelling factor, and *I*_*0MG1*_ is the luminescence intensity at the low-temperature limit, Fig. 6b. This relation enables estimation of the temperature, *T*_*0*_, above which the defect will be thermalized; thus, a higher *T*_*0*_ value means that the defects are stable at higher temperatures. The *T*_*0*_ calculated for the MG1 defect by fitting the data in Fig. 6b is 18.9 K for the NBP:nAg,Er^3+^ sample and 22.2 K for the NBP:nAg sample. The slightly lower *T*_*0*_ value for the sample co-doped with erbium ions and nAg in comparison with the sample doped only with nAg can be attributed to overlapping of the MGR1 and MG1 bands. This overlap can result in imprecision in the deconvolution process when Gaussian shape approximations of both emission bands are applied for NBP:nAg,Er^3+^.

The temperature dependence of the FWHM of the emissions also confirms the different behaviours of both defect centres, where the MGR1-centre fits the CC model and the MG1 does not. In Fig. 6c, the evolution of the FWHM with the square root of temperature, *T*^*1/2*^, is shown for MGR1 (1.078 eV) and MG1 (1.192 eV) emissions in the NBP:nAg,Er^3+^ sample. The dashed lines are theoretical fits to the measured data using the CC model and the following expression:

3 $${\rm{FWHM}}={[8\mathrm{ln}(2){\rm{S}}]}^{1/2}{{\rm{E}}}_{{\rm{ph}}}[\,\coth \,{({{\rm{E}}}_{{\rm{ph}}}/(2{\rm{kT}})]}^{1/2}$$

where k is the Boltzmann constant, T is the temperature, E_ph_ is the energy of the vibration mode of the excited state, and S is the Huang–Rhys factor (see Supplementary Materials). The best fit for the MGR1 FWHM emission was obtained for the Huang–Rhys factor *S* = 2.8 and the energy of the vibration mode of the excited state *E*_*ph*_ = 11.4 meV. The Huang–Rhys factor *S* > 1 indicates the presence of a strong defect-related electron–lattice coupling, which results in a broad Gaussian line shape without any defined structure. The fitted phonon energy, *E*_*ph*_, is much lower than that of the lattice phonons of the glass matrix, which indicates coupling of defect-related electrons with local phonons.

The Frank-Condon shift,

4 $${{\rm{\Delta }}}_{{FC}}={S}\ast {{E}}_{{ph}}$$

is related to the strength of the local electric field around the defect and describes the interaction with local phonons. It results in lower photoluminescence energy than thermal activation energy, *E*_*th*_, of the defect (the difference between the minima of the ground state and excited states), as observed in the CC diagram in Fig. 5. The Frank-Condon shift for the MGR1 defect is equal to 31.9 meV. The thermal energy for the MGR1 defect is estimated as ∼1.11 eV based on the following formula:

5 $${{E}}_{{th}}={{E}}_{{PL}}+{{\rm{\Delta }}}_{{FC}}$$

where *E*_*PL*_ is the luminescence transition energy for the MGR1 emission, which is equal to 1.078 eV.

In contrast to the MGR1-centre emission, the FWHM of the MG1-centre emission increases much more quickly than the CC model predicts, and follows an exponential curve, Fig. 6c. This can be a consequence of (i) stronger electron-phonon coupling than the CC model predicts, and (ii) high sensitivity of the defect energy to the local structural variations, which results in thermal quenching of the defect at lower temperatures. The FWHM at the low-temperature limit of the MG1 band (86 meV) is almost two times broader than that of the MGR1 band (45 meV), which indicates stronger sensitivity of the MG1 defect to any local structural inhomogeneity.

The maximum peak position for both the MGR1 and MG1 emissions moves to lower energies as the temperature increases, which demonstrates a so-called “negative-energy shift”^41,42^, Fig. 6d. The negative shift occurs if the vibrational energy of the excited-state phonon is smaller than the vibrational energy of the ground-state phonon. However, the shift in the MGR1 emission peak energy with respect to the temperature is relatively small in contrast to that of the MG1 emission peak energy, which again confirms its smaller sensitivity to the environment and thus, its higher thermal stability.

## CC model description of metastable defect MGR1

Both of the observed defect-photoluminescence peaks can be switched off by cooling the samples under laser light illumination and switched on by cooling the samples in darkness without illumination. The temperature behaviour of the MGR1 defect can be explained according to the CC model (Fig. 5) with the presence of a metastable state MGR1**.

After cooling in darkness, the MGR1 defect is in the MGR1^0^ ground state. Irradiation of the sample cooled in darkness with a 488 nm laser line excites the erbium ion from the ground state ^4^I_15/2_ to the excited state ^4^F_7/2_, and it can partly excite the surface plasmons in nAg. Therefore, the transfer of the MGR1 defect to the energetically higher state MGR1* is due to the energy transfer from the excited erbium ions or from surface plasmons, as no additional absorption was observed at the excitation energy, Fig. S1. The energy of the 488 nm argon laser line (2.54 eV) is resonant with the ^4^I_15/2_–^4^F_7/2_ Er^3+^ ion transition; thus, the excitation of erbium ions would be expected to be much stronger than the excitation of surface plasmons with an energy lower than the resonant energy (3.06 eV). This is why the primary energy transfer to the MGR1 defect is likely due to energy transfer from one of the Er^3+^ excited states to the MGR1 defect. However there should be also an interaction of erbium ions and nAg since no defect state has been observed in a material doped only with Er^3+^ ions. The transition from the MGR1* excited state to the ground state occurs with emission at 1150 nm (1.078 eV). As the temperature increases, more electrons populate the excited state at the point *T*_q_ where the two state curves cross in the CC diagram (Fig. 5), and electrons can return to the ground state through non-radiative transitions. Under illumination with a 488 nm laser line during the cooling process, the defect centre remains at the MGR1* excited state. At *T* ≈ *T*_t_, when the potential energy curve matches the energy for the metastable state MGR1**, the centre relaxes to the minimum energy state for the metastable configuration, from which the transition to the ground state is impossible. This explains the absence of MGR1 defect-related photoluminescence from the samples cooled under illumination, Fig. 4.

It is currently not possible to fully explain the structure of the metastable defects responsible for the defect emission in the NBP:nAg and NBP:nAg,Er^3+^ nanomaterials; however, preliminary suggestions can be made. The boron-phosphate glass network is formed by PO_4_ and BO_4_ tetrahedra that form chains linked through the covalently bonded bridging oxygen ions (BO). On the other hand, the terminal oxygen ions in the chain, non-bridging oxygen ions (NBO), are doubly bonded to phosphorous^43^. Additionally, phosphate glasses usually have the free hydroxyl (OH) group bonded to the NBO by hydrogen bonding, which is manifested by the absorption at around 3300–3700 cm^−1^ ^44^. We found, however, that the OH group absorption at 3340 cm^−1^ is two times lower for the NBP:nAg sample than for pure NBP, Fig. 2S. This may suggest that part of the NBO can interact with the silver atoms at the Ag nanoparticle surfaces, thus leading to a decreased amount of OH groups attached to the NBO. At the same time, this may lead to the formation of MG1 defects, as the emission at 1150 nm is only observed when Ag nanoparticles are present in the glass.

Electronic transitions from the exited states to the ground state within the 4 f shell of erbium ions can occur only when erbium ions are located in a non-centrosymmetric local field^38^. In oxide glasses, such a field is present when erbium ions are located in the centre of the octahedron formed by oxygen ions^45^. On the other hand, the MGR1 defect only appears in the NBP glass co-doped with erbium ions and nAg. Such a relationship can only exist when both components are located at atomic distance from each other. Thus the oxygen may play the role of electronically bridging erbium ions and silver nanoparticles and the MGR1 defect is created at the interface between erbium octahedra and oxygens bonded to the silver nanoparticles. Although all presented data confirm unambiguously that MGR1 and MG1 are two distinct defects, it is possible that, in the presence of erbium ions, the MG1 defect could be partly transformed into the MGR1 one.

## Conclusions

In conclusion, the new metastable defects emitting NIR light were found in the bulk plasmonic composites doped with silver nanoparticles and co**-**doped with silver nanoparticles and erbium ions. The defect emission could be reversibly photo-quenched to a PL-inactive metastable state by cooling under illumination, and thermally recovered to a PL-active state by heating to room temperature and cooling in the dark. Switching times compared favourably with those previously reported for vitreous systems^14^, and no damage was incurred from the irradiation. To the best of our knowledge, this is the first time that metastable defects have been demonstrated in either passive or active nanoplasmonic materials, and, more generally, in a non-semiconducting material.

Such metastable states in glass matrices doped with silver nanoparticles or co-doped with silver nanoparticles and erbium ions could potentially be used in localized optical data storage systems based on photoluminescence. In this application, data could be written by controlled laser irradiation while the sample is cooled, and only unilluminated regions would be PL-active. Subsequently, the data could be easily erased using the thermal recovery process. This type of material could potentially be used for writing, reading and simple erasing of data in three-dimensions through such methods as direct laser writing^46,47^. However, the defect-related on/off-switching luminescence processes described here were only observed at low temperatures.

## Methods

### Composite Manufacturing

Na_5_B_2_P_3_O_13_ glass rods doped with erbium ions and Ag nanoparticles (nAg) were manufactured by the NPDD method^1^, which utilizes the micro-pulling-down concept. The micro-pulling-down method^48,49^ (µ-PD) was originally invented for the growth of single-crystal fibres, and was then also used for eutectic materials. It has recently been utilized to manufacture metamaterials^50^ and eutectic-based plasmonic nanocomposites^28,51^. The raw materials were prepared as follows: (i) Na_5_B_2_P_3_O_13_ (NBP) glass matrix was prepared from high-purity Na_2_CO_3_ (99.99%), NH_4_H_2_PO_4_ (analytically pure), and H_3_BO_3_ (99.99%) in a 2.5:3:2 molar ratio. The material was mixed in an alumina mortar and synthesised at 850 °C; (ii) Nominally spherical Ag nanoparticles, 20 nm in diameter and of 99.9% purity, were used (by Nanostructured & Amorphous Materials, Inc. Houston, USA); (iii) For samples co-doped with erbium ions, Er^3+^ was introduced into the nanocomposite in the form of erbium oxide, Er_2_O_3_ powder (Alpha Aesar 99,9%); (iv) The final raw material was prepared by mixing appropriate amounts of the powdered glass matrix and the respective nanoparticles and powdered oxide using an alumina mortar and/or a planetary mill. After mixing in the planetary mill, the agglomerates were partially separated, and the particles were pressed onto the surface of glass fragments. After the raw materials were introduced into the crucible (during the solidification process) and heated up, convection was used for further mixing of the material. A commercial μ-PD equipment (Cyberstar) was used for the solidification process. Inductive heating followed and the experiments were carried in nitrogen atmosphere. Platinum crucibles and alumina after heaters were used. The applied pulling rates ranged from 0.5 to 3 mm/min.

### Photoluminescence Characterization

PL spectra were obtained from excitation with a 488 nm Ar^+^ laser line. The diameter of the laser beam spot was approximately 0.5 mm. The laser beam intensity was changed from 0.03 W to 160 W. The backscattering technique was used for collecting the emission signal. The wavelength resolution of the measurements was 1.7 nm at 1 µm (with a photon energy resolution of 2.1 meV). The PL signal was dispersed with a double-grating 0.46 m monochromator and collected using the lock-in technique and a Hamamatsu photomultiplier R5509-72 with an InGaAsP cathode. The samples were mounted on a cold finger of the cryostat operated in a closed-cycle cooling system at the temperature range from 3.6 K to 300 K. It is likely that for such a configuration, the temperature of the sample upon excitation might be considerably different from the measured temperature. Therefore, the real sample temperature was obtained from the calibration curve based on the temperature dependence of the FWHM of the free exciton line (FE) in silicon according to the literature^44^. One can expect a small difference between the real and estimated temperatures (obtained from the calibration curve of the sample) for temperatures lower than 20 K owing to the difference between the thermal coefficients of silicon and glass.

### Absorption Spectra

The absorption spectra were recorded at 300 K and 14 K by using double-beam Cary 500 and Fourier transform Vertex 80 v spectrophotometers. Low-temperature absorption measurements were performed by using a closed-cycle cooling system at 14 K. Values for the absorption/extinction coefficient κ(λ) of NBP glasses doped with Er^3+^ were calculated according to the following expression:

6 $$\alpha (\lambda )=\frac{2.3026\ast \mathrm{log}({I}_{o}(\lambda )/I(\lambda ))}{d}$$

where log(I_o_/I)is the optical density, d is the sample thickness and λ is the wavelength.

## Electronic supplementary material

Supplementary file
