# Supplementary material for: Optically-active metastable defects in volumetric nanoplasmonic composites
Source: Sci Rep. 2018 Sep 7;8:13425. doi: 10.1038/s41598-018-30803-0 (PMC6128831; doi:10.1038/s41598-018-30803-0)
Supplement: Supplementary file 1 — Supplementary file [file 41598_2018_30803_MOESM1_ESM.doc]

Optically-active metastable defects in volumetric nanoplasmonic composites

Marcin Gajc^1^, Hancza B. Surma^1^,Dorota A. Pawlak^1,2*^

^1^Institute of Electronic Materials Technology (ITME), Wolczynska 133, 01-919 Warsaw, Poland

^2^Chemistry Department, University of Warsaw, ul. Pasteura 1, 02-093 Warsaw, Poland

*Corresponding authors: Dorota A. Pawlak

Institute of Electronic Materials Technology

ul. Wolczynska 133

01-919 Warsaw

Poland
Ph.: +48 22 8349949

Fax: +48 22 8349003, +48 22 8645496

E-mail: [Dorota.Pawlak@itme.edu.pl](mailto:Dorota.Pawlak@itme.edu.pl)
 Barbara.Surma@itme.edu.pl

**Supplementary information**

**Figure 1S.** Extinction spectra measured at 15 K for the NBP:nAg,Er^3+^ sample cooled in darkness and under illumination.

**Configurational coordinate model**

Williams and co-workers[[1]](#endnote-2) presented the configurational coordinate model for the first time by associating the activator and coactivator with the nature of the acceptor and donor which occupy the nearest lattice sites in the crystal. The electronic transition from the excited or ground state of the donor to the ground state of the acceptor gives rise to the luminescence. Owing to a Coulombic attraction between the activator and coactivator, a stable localized centre is formed that has the characteristics of a molecule embedded in the lattice (for a crystal, glass or amorphous material).[[2]](#endnote-3)

Any atomic structure can be also treated as a stable localised centre when the transitions between its ground state and the exited state, known as vibrational-electronic transitions, are not only the pure electronic ones but are accompanied by the absorption or emission of phonons. It takes place when a strong local field is usually formed around these types of defects, and the atomic positions of the ground and excited states are not the same in the defect’s equilibrium state. If a vibrational-electronic transition occurs between two charge states of the “molecule”, the molecule moves to a new vibrational level during the electronic transition and the atomic structure relaxes to its equilibrium position along with emission or annihilation of one or more local phonons. Sometimes, due to the external conditions such as temperature and/or illumination, thermal collapse may occur with the system passing from the exited state to another metastable state (trap) associated with the same defect from which an optical transition is not allowed. The cross-over energy *E_T_* is required either for thermal ionization or for collapse of the system. The equilibrium value of the coordinate for an excited state from which emission is allowed is *q_em_* while the additional energy *E_T_* places the system in a vibrational state *q_em_+q_m_* where *q_m_* is a critical increase (or decrease) in the configurational coordinate required for empting the trap^3^

According to the CC model, the shift of the peak emission with temperature E_em_(T)-E_em_(0) is proportional to (ν_g_^2^-ν_e_^2^)/ν_e_^2^ [[3]](#endnote-4) where ν_g_, and ν_g_ are frequencies of the vibrations associated with the centres of the ground and exited state levels, respectively. This shows that the emission peak shifts towards higher energy with increasing temperature when the frequency of the ground state is higher than excited state (ν_g_>ν_e_) (“positive shift”) and when  ν_g_<ν_e_ it shifts towards lower energy (“negative shift”). The configurational coordinate (CC) diagram is often very useful in analysing the energies in the different charge states of such defects.[[4]](#endnote-5) It assumes that the energy of the defect or impurity depends on its atomic configuration. A single coordinate (configurational coordinate q) is representative of the changes in the spatial configuration of the centre owing to effects such as the magnitude of the breathing relaxation or the off-displacement of the defect/impurity after its charge state changes. This means that the coordinate value for which the minimum of the total energy (defect + lattice) occurs can be different in different charge states. The energy of the centre in each individual charge state can be plotted as a function of this coordinate. For small displacements, elastic restoring forces mean that the energy has a quadratic dependence on the displacement; therefore, the energy vs. coordinate plot is a parabolic function. According to the Franck-Condon principle, optical excitation and recombination processes between the ground and excited states occur “vertically”, and the processes occur through a transition from the minimum of one curve to a portion of the side of the second curve. The electron-phonon coupling causes the centre to relax to the minimum energy state. Plotting the dependence of the energy of the centre on the coordinate for the ground and excited states by using experimentally obtained values for the absorption and emission processes, one can obtain information about the electron-phonon coupling. The intensity of the emission that involves p phonons, *I_p_*, is given by the Poisson distribution (1):

(1)

where *S* is the Huang-Rhys factor and p is the number of phonons involved in the transition

The Huang–Rhys factor *S*>1 indicates that a strong electron-lattice coupling exists, and therefore, the emission band has a broad Gaussian line shape without any defined structure. According to the quantum-mechanical modification of this model^3^, the theoretical temperature dependence of full width at half maximum, (FWHM=Γ(T)), follows the equation below:

$\Gamma(T)=(8\ln2)^{1/2}S^{1/2}h\nu_{ph}[\coth(h\nu_{ph}/2kT)]^{1/2}=B\left[\coth(hv_{ph}/2kT)\right]^{1/2}$ (2)

where $h\nu_{ph}$ is the energy of the vibrational mode of the excited state, *S* is the Huang-Rhys factor that represents the electron-phonon coupling strength, *k* is the Boltzmann constant and *T* is the absolute temperature.

From the fitting procedure of the experimentally measured temperature dependence of the curves’ FWHM one can obtain two parameters, S and *hν_ph_*, and calculate the Franck-Condon parameter $\Delta_{FC}=S*E_{ph}$. The change in the integrated intensity of the emission band, versus temperature, fitted with the Arrhenius formula:


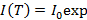
(ΔE/kT)

allows one to obtain ΔE, the energy of thermal quenching of the defect. All these parameters were used to construct the CC model for MGR1 defect.

For amorphous or glass structures, the phonon broadening, *Γ*, is relatively large and has no discrete features. This is due to the disorder of the glassy host. The local environment of the molecular impurity changes from site to site, which results in a site-dependent shift in the vibrational frequency.[[5]](#endnote-6) In addition to phonon broadening, temperature-independent broadening of the emission line is introduced to an extent by crystal imperfections, strains and impurities. The observed emission broadening is a superposition of all these effects.

The CC model also explains the behaviour of defects that can change their atomic configuration under pressure, illumination or temperature. Such defects can also show so-called bistability (metastability), and they are often referred to as DX or AX centres, e.g., silicon in GaAs[[6]](#endnote-7) and oxygen in Al*_x_*Ga_1-_*_x_*As.[[7]](#endnote-8) Optically induced localized paramagnetic states have also been observed in several chalcogenic glasses and amorphous arsenic for *T*< 80K.[[8]](#endnote-9)


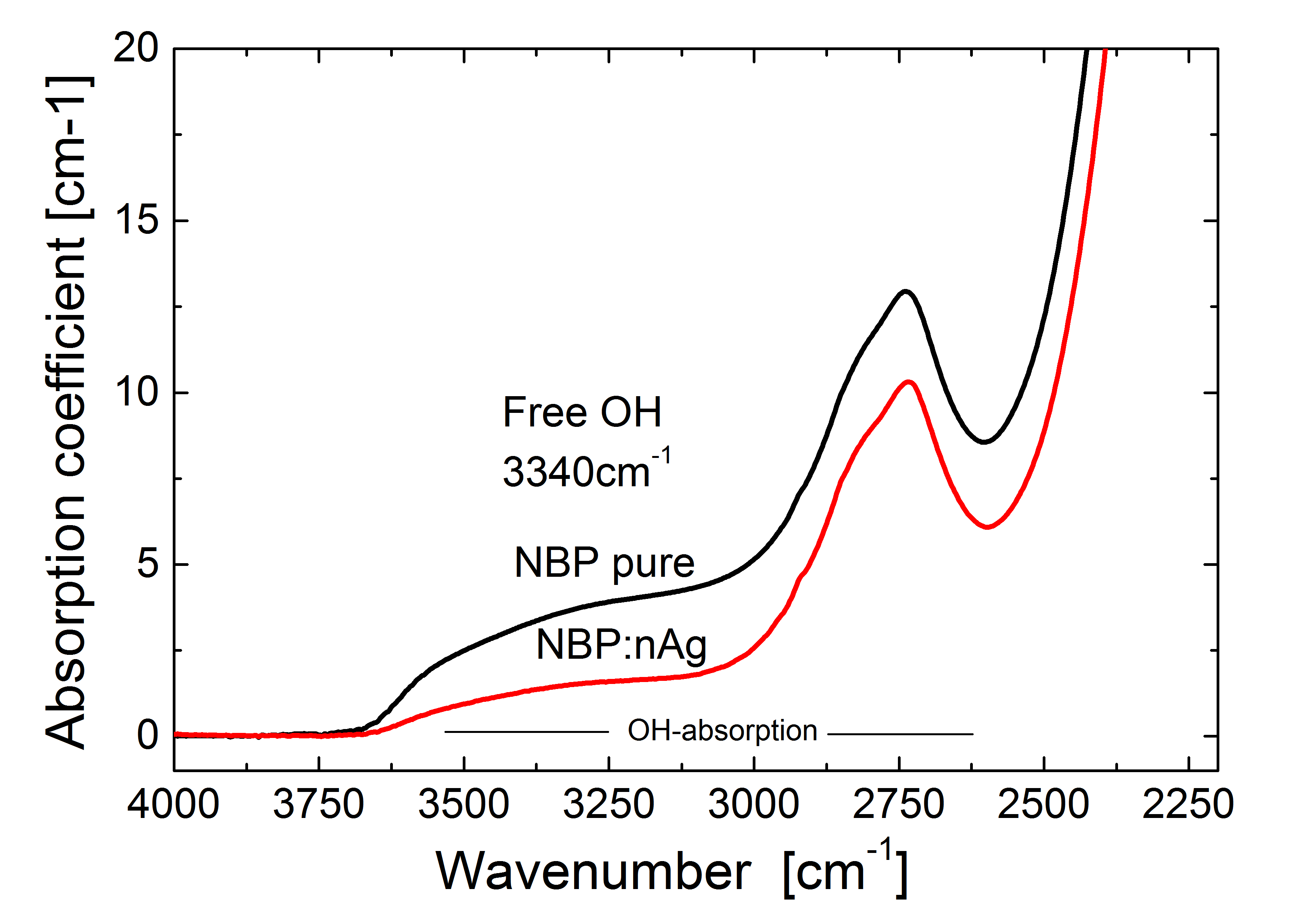


**Figure 2S.** The free hydroxyl group absorption in pure NBP and NBP:nAg glass samples.

1. . J.S. Prener and F.E. Williams *J. Electochem.Soc.***103**,342 (1956). [↑](#endnote-ref-2)
2. . T. Koda and S. Shionoya *Phys. Rev*. **136**, 136, A541 (1964). [↑](#endnote-ref-3)
3. . S.Shinoya, T. Koda, K. Era, and H.Fujiwara, *J. Phys. Soc Jpn* **19**,1157 (1964). [↑](#endnote-ref-4)
4. . C.C. Klik and J. Schulman *Solid State Phys*. **5**,100 (1957). [↑](#endnote-ref-5)
5. . J.R. Engholm, U. Happek, A.J. Sievers *Chem. Phys. Lett*. **249**, 387 (1996). [↑](#endnote-ref-6)
6. . R.C Newman *Semicond. Sci. Technol*. **9**,1749 (1994). [↑](#endnote-ref-7)
7. . Chris G.Van de Walle, *Phys.Rev.B.* **57**, 57 (1988). [↑](#endnote-ref-8)
8. . S. G. Bishop, U. Strom, P. C. Taylor, *Phys. Rev. B* **15**, 2278 (1977). [↑](#endnote-ref-9)
